# Statistical Approaches for the Study of Cognitive and Brain Aging

**DOI:** 10.3389/fnagi.2016.00176

**Published:** 2016-07-19

**Authors:** Huaihou Chen, Bingxin Zhao, Guanqun Cao, Eric C. Proges, Andrew O'Shea, Adam J. Woods, Ronald A. Cohen

**Affiliations:** ^1^Department of Biostatistics, University of FloridaGainesville, FL, USA; ^2^Department of Aging and Geriatric Research, Center for Cognitive Aging and Memory, Institute on Aging, McKnight Brain Institute, University of FloridaGainesville, FL, USA; ^3^Department of Mathematics and Statistics, Auburn UniversityAuburn, AL, USA

**Keywords:** semiparametric model, graphical model, penalized regression methods, structural covariance, functional connectivity

## Abstract

Neuroimaging studies of cognitive and brain aging often yield massive datasets that create many analytic and statistical challenges. In this paper, we discuss and address several limitations in the existing work. (1) Linear models are often used to model the age effects on neuroimaging markers, which may be inadequate in capturing the potential nonlinear age effects. (2) Marginal correlations are often used in brain network analysis, which are not efficient in characterizing a complex brain network. (3) Due to the challenge of high-dimensionality, only a small subset of the regional neuroimaging markers is considered in a prediction model, which could miss important regional markers. To overcome those obstacles, we introduce several advanced statistical methods for analyzing data from cognitive and brain aging studies. Specifically, we introduce semiparametric models for modeling age effects, graphical models for brain network analysis, and penalized regression methods for selecting the most important markers in predicting cognitive outcomes. We illustrate these methods using the healthy aging data from the Active Brain Study.

## Introduction

Multimodal neuroimaging collected in cognitive aging studies provides a noninvasive way to investigate brain changes in structure, function, and metabolism as people age, and thus helps us to understand age-related cognitive changes. However, the high-dimensionality and complex structure of those multimodal neuroimaging data raise statistical challenges. Additionally, the age range is large in aging studies and very often the age effects may not be linear in the large age interval. For instance, participant's age ranges from 50 to 90 in the Active Brain Study, a successful aging cohort. To efficiently analyze those data, there is a strong need for introduction of advanced statistical methods. We will elaborate on the limitations of several existing methods and introduce three advanced statistical methods in sequence.

First, age is a complex variable and often has a nonlinear effect on the outcomes of interest. In developmental studies, flexible semiparametric models have been well used, because it is well-known that growth curves are nonlinear. However, in aging studies, linear or quadratic models are often used to characterize age-related changes. Although a majority of aging research treats aging as a linear process (constant rate of change) and linear models are often considered the gold standard method for evaluating aging effects, this approach may not be the most effective method for representing the complexity of aging data. For instance, Raz et al. (2010) used linear mixed effects models to characterize the age-related brain structural changes in a longitudinal neuroimaging study with 76 participants whose age ranges from 49 to 85. Similarly, Resnick et al. (2003) also used linear mixed effects models to show the age-related brain structural changes in the longitudinal Baltimore study. However, as noted in Fjell et al. (2010), Gogtay et al. (2004), and Thompson et al. (2011); brain structure may show complex age-related nonlinear changes, and could be misspecified by a linear or quadratic model. We have shown that misspecified linear models can result in biased estimates and low powers in statistical tests (Chen et al., 2012). As a nonparametric method, a spline model is recommended for its flexibility and robustness. To accurately model the age trajectories of the neuroimaging markers, we will introduce a spline-based semiparametric model in the methods section and illustrate these methods using the structural neuroimaging data from the Active Brain Study in the example section. The semiparametric model excels at determining rates of global and regional brain atrophy and identifying vulnerable regions of interest (ROIs) susceptible to aging.

Second, marginal correlations are often used in brain network analyses. For example, structural covariance was studied in Mechelli et al. (2005) and Alexander-Bloch et al. (2013), which may be related to structural and functional connectivity. There is also a large literature on Pearson correlation based functional connectivity analysis, where the correlation between two functional magnetic resonance imaging (fMRI) time series [that is the blood-oxygen-level dependent (BOLD) signal] is computed. However, marginal correlation between two brain ROIs is indirect and weak in the sense that all the components in a system are correlated to some degree. Two regions can be indirectly associated with each other due to their correlation with a third region. Moreover, when the number of ROIs is large, the sample covariance/correlation matrix is unstable, as the number of parameters increases quadratically with the number of ROIs. Alternatively, graphical models are attractive for inferring brain connectivity due to their advantages over conventional marginal correlation based analysis (Lauritzen, 1996; Yuan and Lin, 2007; Koller and Friedman, 2009). Graphical models can generate either partial correlations or a binary undirected graph. Sparse penalty is used to regularize the loglikelihood function and make the solution robust. Partial correlation is a desirable measure, as it quantifies the conditional association between two ROIs given the rest of ROIs. Partial correlation can be interpreted as the adjusted correlation. Preliminary applications to neuroimaging data can be found in Salvador et al. (2005), Valdés-Sosa et al. (2005), and Smith (2012). In the methods section, we will introduce two graphical methods for brain network analysis. We will apply these methods to the cortical thickness data from the Active Brain Study for building cortical networks.

Third, the high dimensional neuroimaging markers may provide informative early signs of age-related cognitive and functional decline. For example, brain atrophy in the basal ganglia, hippocampus, and prefrontal areas often precedes the clinical diagnosis of cognitive impairment (Amieva et al., 2005; Grober et al., 2008; Jedynak et al., 2012). It is of great interest to select the informative neuroimaging markers for predicting cognitive decline. However, the high-dimensionality of the neuroimaging markers posit challenges on how to efficiently pick up the informative subset of the markers. Traditional backward or forward variable selection methods are computationally inefficient given the large number of neuroimaging markers. Also neuroimaging markers are often highly correlated with each other. The unpenalized least square based estimates often suffer from high variability or instability (that is with large variance). Moreover, when the number of neuroimaging markers is larger than the sample size, the design matrix is singular and not invertible, and thus there is no unique estimate. In contrast, penalized regression methods can lead to stable solutions and are computationally efficient by using advanced algorithms (Tibshirani, 1996; Fan and Li, 2001; Zou, 2006; Meinshausen and Bühlmann, 2010). Penalized regression methods can simultaneously select and estimate the effects of the predictors. The variable selection is achieved by the sparsity penalty. In the methods section, we will introduce penalized regression methods for selecting the optimal subset of neuroimaging biomarkers for predicting cognitive outcomes. We will illustrate those methods using structural neuroimaging and cognitive data from the Active Brain Study.

The rest of the paper is structured as follows. In the methods section, we introduce the three sets of methods including the spline-based semiparametric model, graphical models, and penalized regression methods. In the examples section, we apply those methods to the data from the Active brain study. We end our paper with general discussions.

## Methods

### Semiparametric models and methods

We first introduce some notations. Let *n* be the number of subjects and let *R* be the number of ROIs. For the *i*th participant, denote *t*_*i*_ as the age, denote *Y*_*ir*_ as the structural/metabolic imaging markers [for instance, volume, fractional anisotropy (FA), myo-inositol (MI)] at the *r*th ROI, and denote ***Z***_*i*_ as other predictors such as education and sex that we want to study. To accurately and efficiently model the age effects, we introduce the following semiparametric model (1) for neuroimaging markers in cross-sectional studies.

(1) $$Y_{ir}=\mu_{r}(t_{i})+Z_{i}\beta_{r}+\epsilon_{ir},\;i=1,\cdots ,n,\;r=1,\cdots ,R,$$

where μ_*r*_(*t*) is the unspecified aging trajectory for the older people at the *r*th ROI evaluated at age *t*, and **β**_*r*_ are the regression coefficients of the other predictors at the ROI. The measurement errors ϵ_*ir*_ are assumed to be independently and identically distributed and follow a normal distribution $N\left(0,\sigma_{r}^{2}\right)$ with mean zero and variance $\sigma_{r}^{2}$. Model (1) consists of both the nonparametric part μ_*r*_(*t*) and the parametric part ***Z*****β**_*r*_, and thus it is called semiparametric model. The semiparametric model is a parsimonious way to both capture the potential nonlinear age trajectory and investigate the effects of other predictors. Notably, the traditional linear model is a special case of model (1), where the function μ_*r*_(*t*) is specified as a linear function β_0*r*_ + β_1*r*_*t*. Extension of model (1) to longitudinal data case is straightforward, which can be accomplished by either introducing subject-specific random effects or using generalized least square methods (Wood, 2006; Wu and Zhang, 2006).

For estimation, we use spline basis functions to approximate the unspecified function μ_*r*_(*t*). Particularly, we assume μ_*r*_(*t*) = *B*(*t*)**θ**_*r*_, where *B*(*t*) is a set of B-spline basis functions and **θ**_*r*_ is the associated spline coefficients (de Boor, 1978). The B-spline basis functions are piecewise polynomial functions in the age interval. A smoothing penalty $\lambda \int {\left[\mu_{r}^{\prime \prime }\left(t\right)\right]}^{2}dt$ is used to achieve smoothness of the fitted function ${\hat{\mu }}_{r}\left(t\right)$, where $\mu_{r}^{\prime \prime }\left(t\right)$ is the second derivative function of μ_*r*_(*t*), and λ ≥ 0 is a smoothing parameter controlling the degree of smoothness. The tuning parameter λ is crucial for the estimation and inference and is often chosen by data driven methods. By minimizing the penalized log-likelihood function, we can obtain the estimate for these parameters including **θ**_*r*_ and **β**_*r*_. Compared to traditional linear model and methods, spline method offers flexible estimation of these functions. Based on the semiparametric model (1), we will be able to more accurately delineate the aging trajectories and their derivative functions and get unbiased estimates for the parametric part.

The spline-based semiparametric model and methods have been implemented in several R packages (R Core Team, 2012) including the *mgcv* package (Wood, 2006). The *gam* function in *mgcv* can output the estimates and inferential results for both the parametric and nonparametric parts. Specifically, for the parametric part, estimates of the regression coefficients and *p*-values are provided which is similar to a linear regression model. For the nonparametric part, the procedure provides the estimate and pointwise confidence intervals for the estimated function and a *p*-value for testing the function as a constant. The 95% point-wise confidence interval $\left[\mu_{r}^{L}\left(t\right),\mu_{r}^{U}\left(t\right)\right]$ for μ_*r*_(*t*) provides the variability at the age *t* in the *r*th ROI, in addition to the magnitude. The first derivative function of μ_*r*_(*t*) indicate the rate of brain atrophy, where in the linear case is the slope of the line. The first derivative functions are can be easily obtained using *B*′(*t*)θ, where *B*′(*t*) are the first derivative functions of the B-spline basis functions. Based on the first derivative functions of the aging trajectories, ROIs/markers show early atrophy/abnormality could be candidate biomarkers for early diagnosis of diseases. We adjust for multiple comparison by controlling the *false discovery rate* (FDR) (Benjamini and Hochberg, 1995; Benjamini and Heller, 2007).

Remark 1 Misspecified linear models could introduce bias for the estimates of μ_*r*_(*t*) and **β**_*r*_, that is for both the nonparametric and parametric parts.

Remark 2 To achieve good approximations of these unspecified functions, enough number of basis functions should be used for the penalized splines. If the procedure leads to an oversmooth case, one can fit a regression cubic spline with fixed number of knots without penalty, thus the degree of freedom is fixed.

Remark 3 Computing time is not a concern for ROI-level data. Some statistical packages such as the *vows* have implemented massive parallel algorithm for voxel-level data (Reiss et al., 2014).

### Graphical model and methods

We first define a graph *G* = (*V, E*), where *V* is a set of vertices/nodes, and *E* is a set of edges connecting pairs of nodes in *V*. An adjacency matrix of a graph is a binary matrix indicating the connection between the nodes. We introduce graphical models for brain structural and functional network analysis. In the past decade, *Gaussian graphical model* (GGM) has been a hot topic in statistics as a tool for complex system analysis. The GGM has many advantages over the traditional marginal correlation based analysis including resulting in partial correlations, i.e., direct dependency/independence, and sparse networks. Let ***Y*** be an *R*-dimensional random variable following a multivariate Gaussian distribution *N*(**μ**, ***G***^−1^) with mean **μ** and covariance ***G***^−1^. ***G*** is a precision matrix (inverse covariance), and the *i, j*th component of ***G***, *g*_*rs*_ = 0 indicates *conditional independence* between ROIs *r* and *s* given all the other ROIs {1, ⋯, *R*}∕{*r, s*}. The partial correlation between ROIs *r* and *s* is defined as $\rho_{rs}=-g_{rs}∕\sqrt{g_{rr}g_{ss}}$ (Lauritzen, 1996). We obtain a sparse graph by minimizing the following penalized loglikelihood function (Yuan and Lin, 2007):

(2) $$\underset{{}_{G\in 𝔾}}{\arg\min}-\log|G|+\frac{1}{n}\sum_{i=1}^{n}{\left(Y_{i}-\mu \right)}^{T}G(Y_{i}-\mu )+\lambda \sum_{r\neq s}|g_{rs}|,$$

where argmin stands for argument of the minimum, 𝔾 is the set of *R* × *R* positive definite matrices, and λ ≥ 0 is the tuning parameter chosen by a data-driven method. The lasso penalty (Tibshirani, 1996) is used to regularize the loglikelihood function and achieve a sparse solution. This method is often called graphical lasso (glasso) in the statistical literature. Along the same line, Meinshausen and Bühlmann (2006) proposed the node-wise regression based approach for obtaining a binary graph. Both the glasso and the node-wise regression methods have been implemented in the R package *huge* with computational efficient algorithms (Zhao et al., 2012). The *huge* function in the *huge* package can provide estimate for the precision matrix or adjacency matrix of an undirected graph. The stability selection method (Meinshausen and Bühlmann, 2010) is preferred for the selection of the tuning parameter, which controls the sparsity of the estimated precision/adjacency matrix. A large tuning parameter will penalize the loglikelihood function heavily and shrink the small elements in the precision matrix/regression coefficients to zero, while a smaller tuning parameter will barely penalize the loglikelihood function and thus leads to many tiny elements in the precision matrix/regression coefficients.

Once the graph is obtained, graph summary statistics such as centrality measures and clustering coefficient can be computed. For visualizing and summarizing graphical objects, the R package *igraph* provides a set of sophisticated tools (Csardi and Nepusz, 2006).

### Penalized regression methods

To utilize high-dimensional markers for predicting cognitive outcomes, we introduce penalized regression methods for linear models. Penalized regression methods can reduce the dimensionality of the predictors by automatically selecting the optimal subset. The variable selection is achieved by a sparsity penalty such as lasso (Tibshirani, 1996), adaptive lasso (Zou, 2006), elastic net (Zou, 2006), SCAD (Fan and Li, 2001), or by stability method (Meinshausen and Bühlmann, 2010). For the *i*th participant, let *Y*_*i*_ be the cognitive outcome, and let ***X***_*i*_ be the stacked *p* × 1 vector of neuroimaging markers and other covariates. We consider the following linear model and penalized method.

(3) $$Y_{i}=X_{i}\beta +\epsilon_{i},\;i=1,\cdots ,n,$$

(4) $$\beta =\arg\underset{\beta \in {\mathbb{R}}^{p}}{\min}\sum_{i=1}^{n}{\left(Y_{i}-X_{i}\beta \right)}^{2}+\lambda \phi (|\beta |),$$

where **β** are the coefficients for neuroimaging markers and covariates, and ϕ(.) is a penalty function of the regression coefficients **β**. By minimizing the penalized least squares (4), we can obtain the penalized estimator $\hat{\text{β}}$. The sparsity penalty shrinkages those small regression coefficients to zeros, thus the procedure automatically leads to a subset of the predictors. If ${\hat{\beta }}_{j}=0$, then the *j*th predictor *X*_*j*_ is not selected. The sparsity of the estimate is controlled by the tuning parameter λ ≥ 0, which is usually chosen by data driven methods such as cross-validation or generalized cross-validation.

Thanks to the implementation of efficient algorithms, current software package can handle thousands of predictors simultaneously for a medium sample size such as *n* = 80. In general the computational time is moderate and depends on the size of the data that is the sample size *n* and the number of predictors *p*. One of the popular R package *glmnet* has implemented a few penalized methods including lasso and elastic net. The *glmnet* function in the *glmnet* package provides all the coefficient solution paths as functions of the tuning parameter λ. To get the optimal solution, the user needs to use the cross-validation method to select the optimal tuning parameter with the smallest mean squared error (MSE).

Remark 4 The penalized regression methods are applicable to generalized outcomes including binary and count data as well. For example, we can use penalized logistic regression methods to select informative neuroimaging markers in predicting risk of mild cognitive impairment (MCI).

Remark 5 Because the penalty shrinkages those regression coefficients toward to zero according to their magnitude, large differences in the original scale of those predictors can mess up the selection. Therefore, it is recommended to standardize the predictors and make all the variables in the same scale.

## Examples: The active brain study

We illustrate the introduced methods using the data from the Active Brain Study. The aim of the study is to investigate the brain changes associated with age-related cognitive decline via multimodal neuroimaging. We consider *n* = 114 participants with structural imaging. Among them 68% are female. The mean age of the sample is 72.3 with the standard deviation (SD) 10. Those participants are well educated as can be seen from the mean education years = 16.2 (*SD* = 2.6). They also show high cognitive performance with mean Montreal Cognitive Assessment (MoCA) score 25.7 (*SD* = 2.6). The structural imaging was processed using standard procedures implemented in Freesurfer version 5.3 (Dale et al., 1999; Fischl et al., 2002). For a more detailed description of the Freesurfer processing methods used by our group see Szymkowicz et al. (2016). Brain volumetric indices including regional and global volumes of cortical and subcortical structures as well as cortical thickness were generated. Particularly, we used the anatomical cortical parcellation in Desikan et al. (2006), which generated 34 ROIs in each hemisphere. Similarly, the subcortical segmentation of a brain volume is based on the existence of an atlas containing probabilistic information on the location of structures (Fischl et al., 2002).

### Aging-related trajectories of brain regional volumes and areas

We are interested in delineating the aging trajectories for the regional volumes and areas, while adjusting for sex, education, and the total intracranial volume (ICV). For normalization purpose, the regional volumes are divided by the ICV. To check the nonlinearity of the age trajectories of the regional volumes and areas, we first applied the loess (locally weighted scatterplot smoothing) method using the R function *loess*, which is a popular exploratory tool for checking nonlinear pattern. As a lot of ROIs show nonlinear age trajectories of brain regional volumes, we fit a semiparametric model for the normalized volume at each ROI with nonparametric age trajectory and parametric effects for sex and education using the *gam* function in the *mgcv* package. Similarly, we fit a semiparametric model for the area at each ROI with nonparametric age trajectory and parametric effects for sex, education and ICV. Penalized cubic B-splines with 10 basis functions are used to fit the age trajectories. The restricted maximum likelihood (REML, Reiss and Todd Ogden, 2009) method is used to select the tuning parameters. For comparison, we also fit linear and quadratic models for the age trajectories. The quadratic age term is centered to achieve robustness. Alternatively, orthogonal polynomial model can be used to avoid multicollinearity problem.

**Figure 1:**
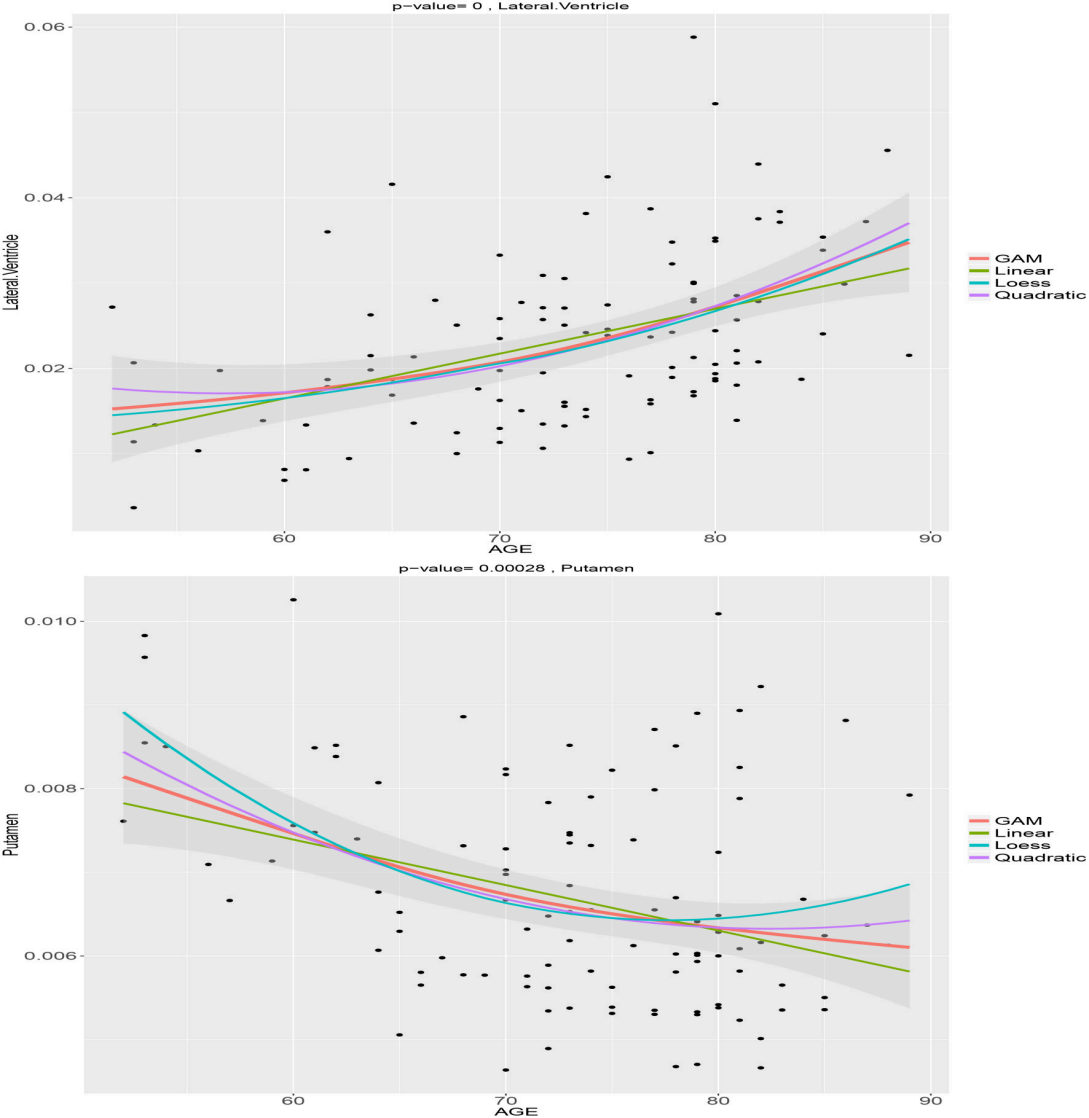
**Age trajectories of the normalized lateral ventricle and putamen volumes using the semiparametric model, loess fit, linear, and quadratic regression models**.

We choose the normalized volume of the lateral ventricle and putamen for illustration. Figure 1 shows the estimated age trajectories (the solid lines) using different methods, and the 95% pointwise confidence intervals (the shaded area) for the B-spline fits. The lateral ventricle displays considerable expansion especially after age 70, while the putamen shows a large amount of decline especially before age 75. Both the lateral ventricle expansion and the putamen volume shrinkage indicate brain atrophy as people age. We notice that both the loess and the semiparametric fits indicate nonlinear age patterns. As displayed in Figure 1, linear models are not flexible enough to capture the nonlinear age trajectories. Linear models assume the rate of age-related change is constant as people age, which may not be true for all the ROIs. The deviation of the linear fits from the semiparmetric model fitted curves are large in the two ends and the middle part of the interval, that is less than 60, greater than 80, and around 70. The quadratic age trajectories show agreement with the B-spline fits around the middle of the age interval [60, 80], but not in the two ends either. The loess fits are exploratory without adjusting for sex and education. Interestingly the B-spline fits agree with the loess fit for the lateral ventricle volume but not the putamen volume.

Overall, age has significant effects on almost all of the cortical and subcortical regional volumes in both hemispheres after FDR correction. Particularly, the cortical frontal, temporal, parietal, occipital, cingulate lobes are significantly impacted by aging except the left caudal anterior cingulate, bilateral entorhinal, pericalcarine, and frontal pole. The insula shows a marginally significant age effect. The ventricle, subcortical regions, and corpus callosum are significant except for the bilateral caudate. Our findings are consistent with the literature that as people age, the brain regional volumes shrink, while the ventricle system and CSF considerably expand. We also observe that older females tend to have less brain atrophy compared to older males after FDR correction. Education does not have a significant effect on any of those regional volumes after FDR correction. Additionally, age shows similar effects on the cortical regional areas. However, after adjusting for the ICV, neither sex nor education has an effect on the cortical regional areas.

In summary, the linear/quadratic model due to its parametric nature, may not be flexible enough to capture age-related brain changes as people age. A nonparametric/semiparametric model should be used if there is a convincingly nonlinear pattern as suggested by an exploratory loess fit.

### Cortical thickness based cortical network

Structural covariance has been used in the literature for studying cortical networks and patterns of neurodegeneration (Mechelli et al., 2005; Alexander-Bloch et al., 2013). Here, we are interested in applying graphical models to investigate the cortical network using the cortical thickness data from the Active Brain Study. We consider the cortical thickness at 34 ROIs in each of the hemispheres. For summary purpose, we group the 68 cortical ROIs into six lobes including the frontal, temporal, parietal, occipital, cingulate, and insula. We first compute the marginal Pearson correlation for the structural covariance/correlation. We then use the *huge* function to obtain the partial correlation (based on the precision matrix) and a binary undirected graph (or equivalently the adjacency matrix) using the glasso and node-wise regression respectively. The tuning parameters are selected by the stability method (Liu et al., 2010; Meinshausen and Bühlmann, 2010).

**Figure 2:**
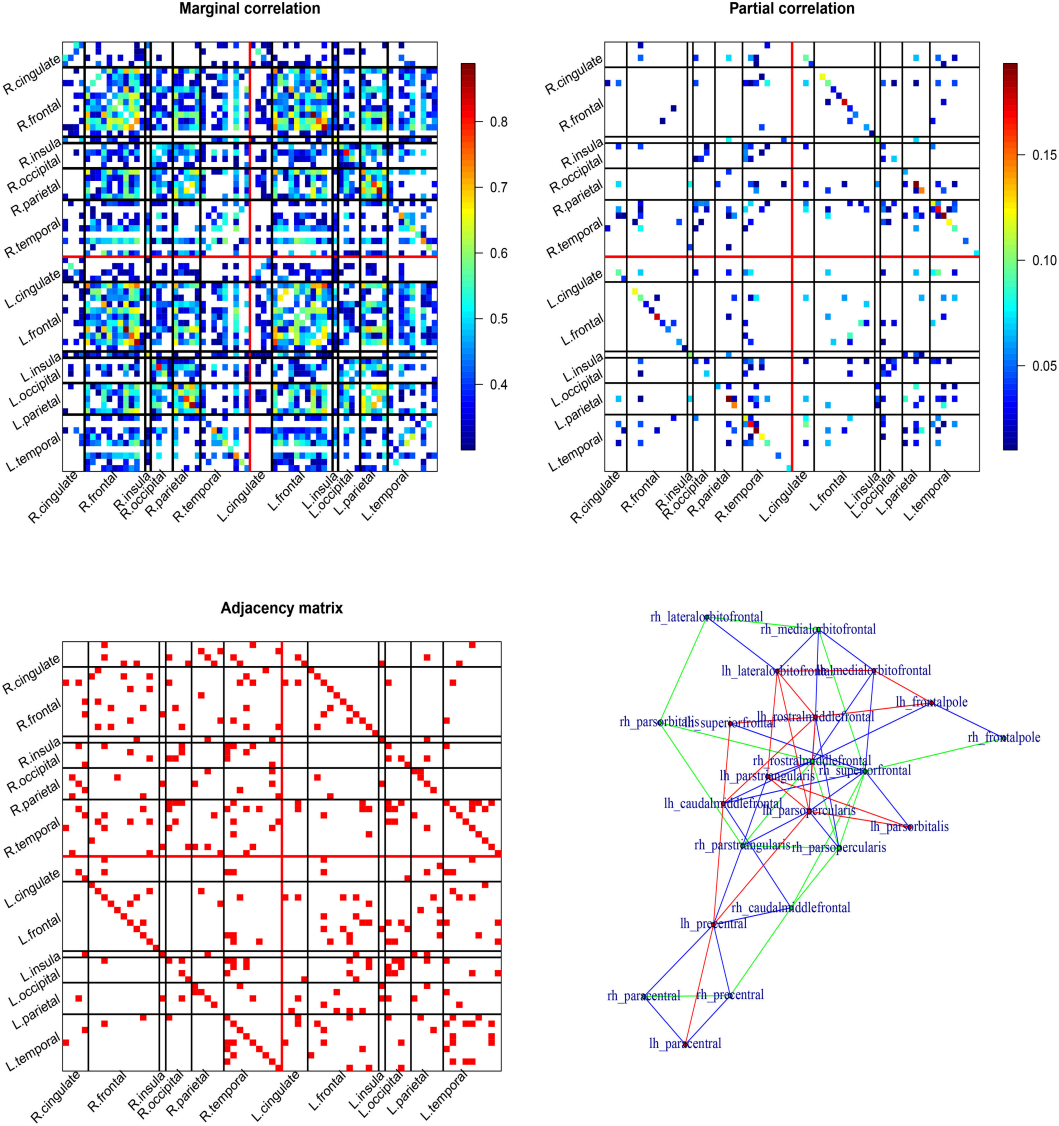
**Cortical thickness based cortical network**. The top two patterns are Pearson's correlation map (left) and partial correlation map (from the Glasso; right) for the cortical network. The bottom two patterns are adjacency matrix of the undirected graph (from the node-wise regression; left) and graphical map of the frontal lobe (right).

The results are summarized in Figure 2. The top two patterns in Figure 2 display the thresholded marginal/partial correlation map for the 68 cortical ROIs (34 ROIs per hemisphere). The bottom two patterns display the undirected graph and the frontal subgraph plotted using function in the *igraph* package. The marginal correlation map is cut by 0.3. The marginal correlation and partial correlation show very different patterns. The range of the marginal correlation is much larger compared to the partial correlation. The two graphical methods share some similarity. The left and right correlation are strong even conditional on all the other ROIs. There are both inter- and intra-hemisphere correlation. Based on the bottom adjacency matrix plot, we observe that the frontal ROIs tend to be conditionally correlated (see also the bottom right panel in Figure 2). Other graph summary statistics can be easily calculated using functions in the *igraph* package such as degree of centrality.

In summary, the marginal correlation and partial correlation map often show different patterns. The interpretation of the two are also different. The marginal correlation between two ROIs does not account for the involvement of other ROIs, while the partial correlation between two ROIs adjusts for other ROIs. Due to the lasso penalty, the partial correlation map and the adjacency matrix are sparse that is some of the partial correlations/elements of the adjacency matrix are estimated to be zeros.

### Predicting MoCA using brain regional volumes

In this section, we aim to select informative brain regional volumes in predicting the cognitive outcome MoCA. We first normalize regional volumes via dividing by the estimated intracranial volume (ICV), then standardize the variables by subtracting the sample mean and divided by sample standard deviation to make the variables comparable. The predictors we consider include the cortical and subcortical regional volumes, age, sex, and education. We used the *glmnet* function in the R package *glmnet* with both lasso and elastic net penalties. We choose the tuning parameters by 10-fold cross-validation.

Table 1 summarizes the selected variables and their coefficients using both penalties. The selected variables include regional volumes from the frontal and temporal lobes, subcortical regions, and demographic variables. Compared to the elastic net penalty, the lasso penalty tends to choose a small subset of correlated predictors. For example, the left pars opercularis (Brodmann area 44) was selected but not the right one. Consistent with the findings in the literature, we found that volumes of subcortical and cortical ROIs including the left accumbens, middle temporal, pars opercularis, temporal pole, right entorhinal, medial-orbito-frontal, pars opercularis are positively associated with MoCA.

**Table 1:** **Selected brain regional volumes and covariates in predicting MoCA**.

	**lh.middle.temporal (temporal lobe)**	**lh.pars.opercularis (frontal lobe)**	**lh.pars.orbitalis (frontal lobe)**	**lh.temporal.pole (temporal lobe)**	**rh.entorhinal (temporal lobe)**	**rh.medial.orbito.frontal (frontal lobe)**
Elastic-net	0.047	0.345	−0.320	0.145	0.188	0.048
LASSO	0.004	0.433	−0.343	0.154	0.193	0.005
	**rh.pars.opercularis (frontal lobe)**	**lh.Accumbens**	**Age**	**Sex (F vs. M)**	**Education**	
Elastic-net	0.031	0.309	−0.231	0.331	0.177	
LASSO	-	0.358	−0.225	0.329	0.177	

**Figure 3:**
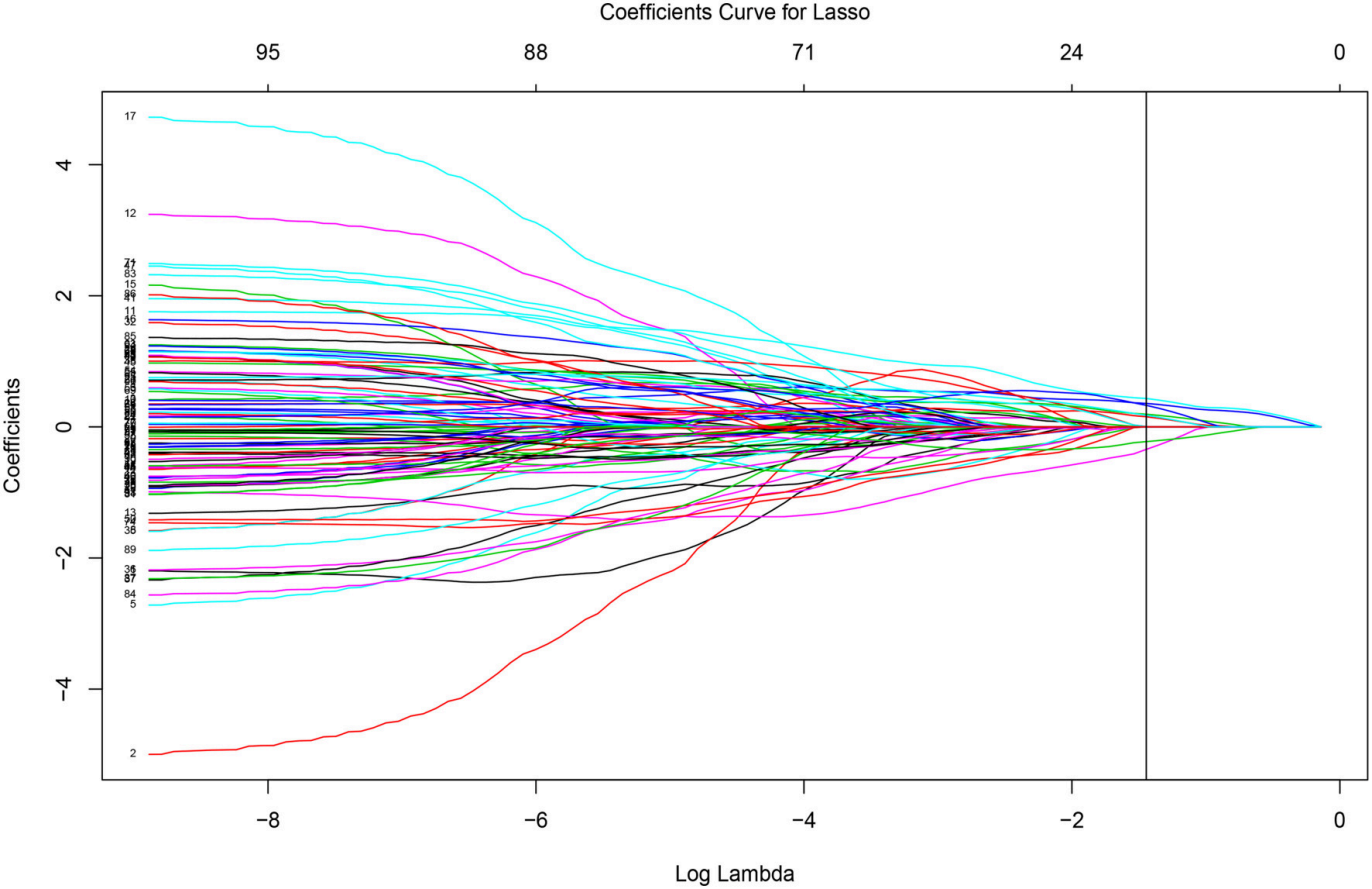
**Solution paths from the penalized regression with lasso penalty**.

The *glmnet* function can output the whole solution path. Figure 3 displays the whole solution path for all the coefficients as functions of the logarithm tuning parameter λ for the lasso penalty. The vertical line corresponds to the optimal λ selected by cross-validation. When the tuning parameter λ is small (that is with less penalization), the magnitudes of the coefficients are large and the variability is large. The traditional least square estimate is similar to the small penalization case, which is not stable. As the tuning parameter increases, the variability of the coefficients declines. The regularization achieves the small variance at the cost of introducing bias. The cross-validation criterion selects the tuning parameter by balancing the variance and bias.

To check the performance of the selected subset of the regional volumes, we refit a model with the volume of the selected ROIs and compare to the model with only age, sex, and education. The selected regional volumes from the elastic net penalty explains additional 19% variance in MoCA, where *R*^2^ increases from 16 to 35%. We conduct an ANOVA test to compare the two models, where the *p*-value is less than 0.001.

## Discussions

Misspecified linear models are not uncommon in the literature, which may lead to biased results and misleading conclusions. Based on our previous neuroimaging analysis (Chen et al., 2014, 2015), linear models may not always be appropriate for characterizing the age-related brain changes, although it is the default method due to its simplicity. In practice, cautions need to be raised for the potential nonlinear age-related changes. To minimize the potential bias, we introduced the spline-based semiparametric models, which are more flexible and able to capture the underlying age trends in the data. Notably a linear model is a special case of the semiparametric model. When the underlying trend is linear, semiparametric model agrees with the linear model. Semiparametric methods have been implemented in many statistical softwares such as R. One of the popular implementations is the *gam* function in the *mgcv* package. The *gam* function provides the estimated curves and inferential results for both the parametric and the nonparametric parts. Extension of the basic semiparametric model (1) has been extensively studied in the past few decades in the statistical literature. More sophisticated models such as varying coefficient models and additive models have also been developed (Wood, 2006; Wu and Zhang, 2006). All these semiparametric methods are scalable and applicable to voxel level data as well. The R package *vows* has implemented semiparametric models for voxel-level data. A parallel algorithm is implemented to speed up the computational procedure.

In the neuroimaging literature, network analysis provides a systematic way to study the brain structural and functional changes. The use of network analysis is a remarkable progress from the pairwise relationship between ROIs. However, researchers often compute the marginal correlation then threshold the correlation matrix to obtain the graph/network. It is well known that sample covariance/correlation matrix is highly instable when the number of ROIs is large. The pairwise nature of the marginal correlations hurts and limits the interpretation of the subsequent network based results. To address the limitation of the marginal correlation, we introduced two Gaussian graphical models, which can generate either partial correlations or an undirected graph. Under the multivariate Gaussian assumption, a zero partial correlation for two ROIs given all the other ROIs is equivalent to conditional independence between the two ROIs. Similarly, for an undirected Gaussian graph, the edges indicate conditional dependence between ROIs. We illustrated the graphical models using the cortical thickness data, where the generated cortical networks may be related to the cortical structural connectivity. The application of graphical model to fMRI for investigating functional connectivity is straightforward, but need to be modified to account for the temporal correlation within each time series. Besides partial correlation in the time domain, there is a few works on correlation measure in the frequency domain such as the total independence (Wen et al., 2012) and partial correlation for multivariate time series (Fried and Didelez, 2005).

For testing the brain network differences between groups such as young vs. older, there are three levels of tests including the edge-level, node-level, and subgraph-level (Nichols and Holmes, 2002; Kim et al., 2014, 2015; Narayan and Allen, 2016). The edge-level testing approach first tests the group differences at the edges one by one, then applies multiple correction for the *p*-values such as FDR correction. The node-level testing method investigates the group differences in graph summary statistics at each node such as degree of centrality. The subgraph-level testing aims to detect either topologically connected cluster difference (Zalesky et al., 2010) or differences in graph overall metrics such as clustering coefficient. The three levels of testing approaches provide complementary ways of testing the brain network differences.

Efficiently and accurately predicting cognitive decline is a central topic in cognitive and brain aging studies. In practice, very often an *a priori* subset of neuroimaging biomarkers are used to predict cognitive outcomes, which are based on the predetermined hypothesis. Hypothesis-driven methods are a recommended way to conduct research that can generate reproducible results. However, by chance, we may miss important neuroimaging markers that could indeed be predictive for cognitive decline. We introduced penalized regression methods for incorporating a large amount of neuroimaging biomarkers in predicting cognitive outcomes, where the number of predictors can be close to or even larger than the number of subjects. These data-driven methods can simultaneously estimate the regression coefficients and select a subset of the high-dimensional predictors. We illustrated those methods using the brain regional volumes in predicting MoCA outcomes. Moreover, these methods are applicable to categorical cognitive impairment outcomes such as a variable with three nominal levels: normal, mild cognitive impairment, and dementia. In addition to the penalized regression methods, some machine learning type of methods such as penalized support vector machine (SVM) can be used for building prediction/classification rule based on high dimensional neuroimaging biomarkers (Zhu et al., 2004; Zhang et al., 2006; Wu and Liu, 2007; Robinson et al., 2015). For long term followup longitudinal studies, penalized mixed effects model can be used to improve the prediction accuracy by incorporating both the individual trajectories and baseline or longitudinal neuroimaging biomarkers (Bondell et al., 2010; Ibrahim et al., 2011).

Neuroimaging data collected in studies of cognitive and brain aging raise statistical and analytic challenges due to the high dimensionality and complex structure. Fortunately, advanced statistical methods developed in the past few decades for high dimensional data and complex structured data could be applied for leveraging the multimodal neuroimaging analysis. These approaches provide a good starting point for analyzing such data. However, there is a strong need for developing new statistical methods that are specific to the multimodal neuroimaging analyses in cognitive and brain aging studies.

## Author contributions

The first two authors HC and BZ conducted the analysis and initiated the paper, while the other five coauthors GC, EP, AO, AW, and RC contributed significant components for the presentations of the models and methods and the general discussions. The last two authors AW and RC are the PIs of the Active Brain Study.

### Conflict of interest statement

The authors declare that the research was conducted in the absence of any commercial or financial relationships that could be construed as a potential conflict of interest.
